# Associations Between Diabetic Foot Deformity and the Intrinsic Foot Muscles: A Systematic Review

**DOI:** 10.1002/jfa2.70068

**Published:** 2025-07-28

**Authors:** Tiffany Hanna, Penelope Latey, Kanchana Ekanayake, Laura Ribarovski, Rebecca McDonald, Jillian Clarke

**Affiliations:** ^1^ Faculty of Medicine and Health School of Health Sciences Discipline of Medical Imaging Science The University of Sydney Sydney Australia; ^2^ Faculty of Medicine and Health School of Health Sciences Discipline of Physiotherapy The University of Sydney Sydney Australia; ^3^ Faculty of Medicine and Health The University Library, Research Services – Publishing Support The University of Sydney Sydney Australia

**Keywords:** diabetes, foot, foot deformity, muscles

## Abstract

**Background:**

Diabetic foot deformities such as claw toes are debilitating complications. It has been generally accepted that this results from atrophy of the intrinsic foot muscles (IFMs), despite this relationship being underexplored. However, recent diagnostic imaging studies are finding some associations between diabetic intrinsic foot muscles and foot deformity. This new information requires a thorough review of the evidence.

**Objectives:**

Our aim is to systematically review the current literature to investigate any associations between diabetic foot deformity and intrinsic foot muscle changes and review their significance.

**Methods:**

Electronic databases Medline, Embase, Scopus, CINAHL, and Web of Science were searched on 6 May 2020 for all years until 2020. The search was re‐run on 26 June 2023 with dates ranging from 2020–2023. Human data studies on adults in English, investigating diabetes, foot deformities and intrinsic foot muscles were included for review. PROSPERO registration was approved on 24 July 2020.

**Results:**

Eleven papers out of 1779 met the inclusion criteria. For the updated search, two papers out of the 226 met inclusion criteria. All were assessed for quality using the STROBE criteria and the Newcastle and Ottawa Scale. Six reported various correlations between intrinsic foot muscles and claw/hammer toe deformity measurements. Three papers reported limited or no associations. Three papers investigated medial column deformity (MCD); two found a significant correlation to intrinsic foot muscle atrophy.

**Conclusion:**

Overall, there is a weak association between diabetic foot deformity and intrinsic foot muscle atrophy and intermuscular adipose tissue infiltration.

## Graphical abstract

Recent diagnostic imaging studies are finding associations between diabetic intrinsic foot muscles and foot deformity. Our aim is to systematically review the current literature to investigate any associations between diabetic foot deformity and intrinsic foot muscle changes. Overall, there is a weak association between diabetic foot deformity and intrinsic foot muscle atrophy and intermuscular adipose tissue infiltration.

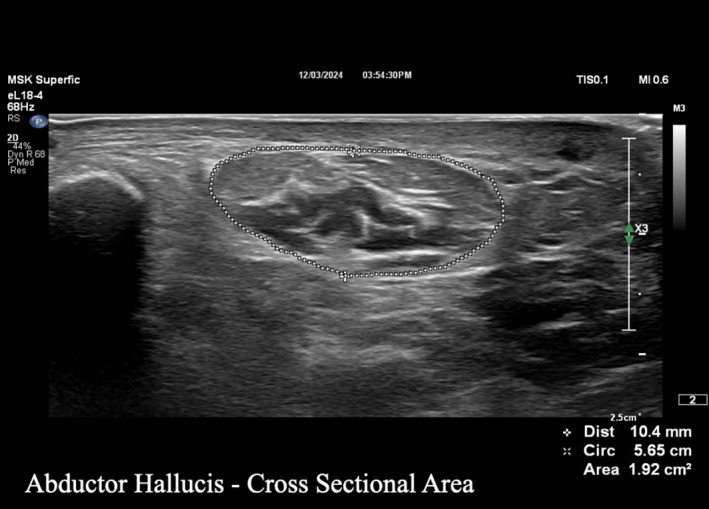

## 1 Introduction

The global prevalence of diabetes was estimated to be 588.7 million individuals in 2024 [1], including 2 million Australians in 2024 [2]. Diabetic peripheral neuropathy (DPN) is the presence of peripheral nerve dysfunction, affecting up to 50% of adult diabetics and is associated with lower limb complications [3] including ulcerations, trauma and ischaemia, which are leading causes of infection, hospitalisation and amputation [4].

The intrinsic foot muscles (IFMs) play an integral role in foot stability, important in diabetic feet [5]. Muscle weakness and atrophy, associated with the severity of DPN, is due to progressive nerve axonal degeneration [6]. Compounding this, muscle quality is also degraded by excessive intermuscular adipose tissue infiltration (IMAT), reducing strength, muscle power and physical function [7].

Further complications are foot deformities including Charcot arthropathy, pes planus, hallux valgus, prominent metatarsal heads and claw and hammer toes [8], with claw toes being the most common. Claw toes are defined as hyperextension of the metatarsophalangeal joint (MTPJ) with flexed proximal interphalangeal joint (PIPJ) and distal interphalangeal joints (DIPJ), whereas hammer toes are characterised by an extended MTPJ, flexed PIPJ joint and a hyperextended DIPJ [9]. Another deformity, medial column deformity (MCD), involves the calcaneus, talus, navicular, cuneiforms and the first to third metatarsals, causing collapse of the arch of the foot [10].

Each year diabetes‐related foot disease affects approximately 50,000 Australians. One of the key risk factors is foot deformity [11]. Concerningly, claw/hammer toes and MCDs are associated with an increased risk of foot ulceration and amputation [12, 13].

The traditionally accepted theory of claw and hammer toe deformities is that of a ‘muscular imbalance’ [9, 14, 15], which is defined as a lack of parity between corresponding agonist and antagonist muscles in a muscle group [16]. A pathological muscle imbalance occurs when the imbalance of the muscles begins to inhibit function and ultimately leads to joint dysfunction [16]. In the case of claw and hammer toes, metatarsophalangeal and interphalangeal joints of the foot become unstable due to weakness of the intrinsic foot muscles [17, 18, 19]. This destabilisation of joints is proposed to cause the proximal phalanx to hyperextend and the distal phalanx to flex resulting in claw or hammer toe deformity. Despite this mechanism being widely accepted and referenced in papers to date, there are only observational studies which lack experimental data to validate the theory.

Some imaging studies using magnetic resonance imaging (MRI) and computed tomography (CT) have cast doubt on this theory. Bus et al. [17] found that muscle imbalance, assessed using a muscle atrophy score they developed [13, 17] and measured with a reliable MRI method to distinguish between muscle and fat, was i) not significantly different between neuropathic patients with and without deformity, and ii) not significantly correlated with the degree of toe deformity (*r* = −0.14). However, Bus et al. [13] only report the reliability of this method for IFM atrophy. The reliability of the extrinsic muscle atrophy score was not reported. Further intrinsic muscle atrophy scores were assessed but only intra‐observer rater reliability was established via weighted Kappa.

In contrast, Cheuy et al. [10] found an overall correlation between IFM deterioration and second MTPJ hyperextension for DPN participants with MCD (*r* = −0.51, *p* = 0.01), using plain radiography and MRI. Similarly, Cheuy et al. [20] found that less forefoot lean muscle tissue was associated with greater MTPJ deformity for participants with DPN (*r* = −0.52, *p* < 0.01).

Allan et al.’s [21] literature review reported that IFM atrophy was present in participants with diabetes and correlated with DPN. Yet, IFM atrophy did not appear to be closely correlated with the incidence of toe deformity in diabetes [21]. However, more recent papers have also found statistically significant correlations between IFM atrophy and MTPJ extension [19, 22].

From the foregoing, the aims of this review are to:

1. Investigate the associations between diabetic foot deformity and the intrinsic foot muscles.
2. Investigate the methods used and the clinical relevance of investigating the associations between diabetic foot deformity and the intrinsic foot muscles.

## 2 Methods

### 2.1 Protocol and Registration

**FIGURE 1:**
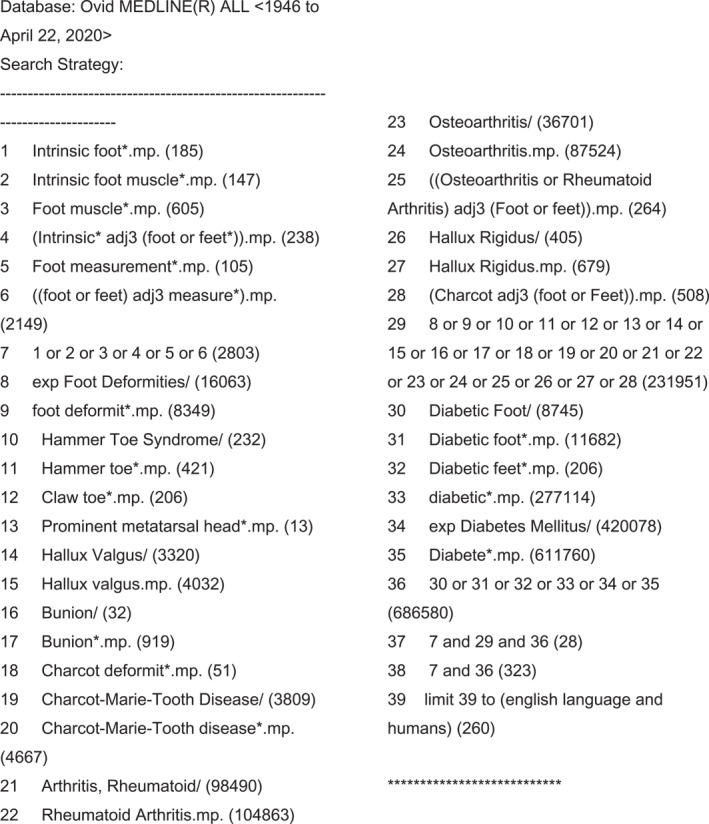
Search strategy.

PROSPERO registration ID: CRD42020193874. Studies were identified by searching electronic databases, online sources and reference lists of articles. Databases were Medline, Embase, Scopus, CINAHL and Web of Science with the search strategy and exclusion criteria demonstrated in Figure 1. All databases were searched on 6 May 2020 and 26 June 2023. Key terms used: diabetes, intrinsic foot muscles and foot deformity. Simultaneous searches were conducted via Google and Google Scholar, using search term ‘Diabetes Intrinsic Foot Muscles Foot Deformity’. Language was English only as no translation services were available. There were no limitations on dates searched. There were no restrictions on study design. Reference lists of included articles were also searched. Results were downloaded for eligibility screening. An additional search was run on 17 January 2025 for papers between 2023 and 2025 that resulted in no additional texts for inclusion.

### 2.2 Study Selection

**FIGURE 2:**
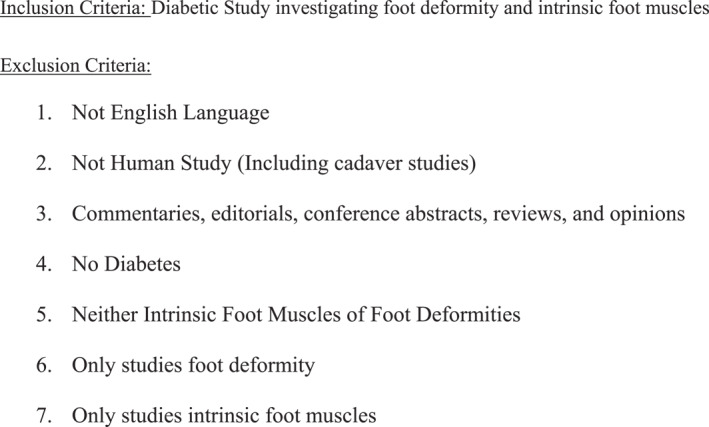
Inclusion and exclusion criteria.

After removal of duplicates, the main reviewer (TH) screened all titles and abstracts, which were also assessed by equal distribution to three independent reviewers (LR, PL and JC). Each reviewer provided a reason for exclusion or inclusion as demonstrated in Figure 2.

Discrepancies were resolved by consensus. Full text eligibility screening was conducted by TH and LR and disagreement was resolved by consensus with a third reviewer (PL or JC). One author was contacted requesting clarification on data and another requesting a full paper of a conference abstract; both responded with the required information.

### 2.3 Quality Assessment

STROBE [23] provided a preliminary quality assessment. The Newcastle–Ottawa scale was used to assess quality and risk of bias [24]. As all included papers were cross‐sectional studies, an adapted version was used [25]. Results are demonstrated in Table 1. Studies scoring a total of eight or seven points were ranked as low risk of bias, six points medium and five points or less indicated a high risk of bias.

**TABLE 1:** Newcastle–Ottawa quality assessment scale.

	Selection (3 stars)			Comparability (2 stars)	Outcome (3 stars)		Total score
Study	Representative of the sample	Sample size	Non‐respondents	The subjects in different outcome groups are comparable. Confounding factors are controlled	Assessment of outcome	Statistical test	
[32]	0	0	0	*	*	*	4
[27]	*	0		*	*	*	4
[17]	0	0	0	*	**	*	4
[10, 18]	*	0	0	**	*	*	5
[20]	0	*	0	**	*	*	5
[29]	0	0	0	*	**	*	5
[28]	*	0	0	*	**	*	5
[19]	*	0	0	**	**	*	6
[26]	*	0	0	**	*	*	5
[30]	0	0	0	*	**	*	4
[33]	0	0	0	*	*	0	2
[22]	*	0	0	*	**	*	5
[31]	*	0	0	*	*	*	4

## 3 Data Collection Process

Data extraction was conducted by the main reviewer (TH). Demographics extracted included study characteristics, type of diabetes, types of deformity, methods of measurements and analysis. Because of the heterogeneity of the data, a meta‐analysis was not possible, so a qualitative analysis was conducted.

## 4 Results

Factors analysed in each study were quality, participant characteristics, methods of measuring neuropathy, IFMs and foot deformity. Statistical results were analysed for any associations between IFMs and foot deformity and their significance.

### 4.1 Included Papers

**FIGURE 3:**
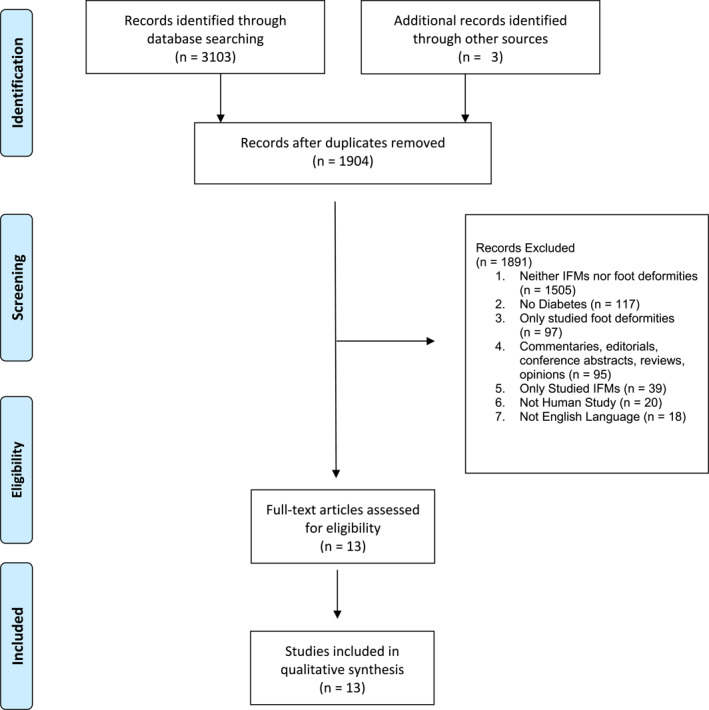
PRISMA flowchart.

A total of 2877 records were retrieved. After removal of duplicates, 1779 were screened. A further three studies were identified by manually searching reference lists of eligible articles. Eleven full papers were deemed eligible for inclusion and data extraction. Two hundred and twenty‐six records were retrieved by the updated 2023 database search; two were deemed eligible for inclusion (Figures 2 and 3).

### 4.2 Quality Assessment

Quality assessment scores are demonstrated in Table 1. Scores ranged from 2 to 6 with a mean of 4. One paper achieved a score of 6 with a medium risk of bias [19]. All other papers scored 5 or below with a high risk of bias. The lowest performing categories were sample size and non‐respondents. One paper provided a justification of sample size and a power calculation [20]. No papers provided insight to participant retainment, dropout and how that data would have been used. The highest performing categories were comparability and assessment of outcomes. All papers controlled for age and sex and most papers assessed data using blinded independent raters.

### 4.3 Participant Characteristics

Participant characteristics are demonstrated in Table 2. The total number of participants from the 13 studies was 1325. Sixty‐five were healthy controls, 13 were diabetics with no peripheral neuropathy and 182 were diabetics with peripheral neuropathy. One thousand and sixty‐five were diabetic participants, but neuropathic status was either not measured or recorded. The sample size of all studies ranged from 2 to 1065 participants with a median of 34. Ages ranged from 27 to 80 years with a control group mean of 53.8 years, diabetes mellitus (DM) with no peripheral neuropathy with the mean of 56.1 years, and diabetes with peripheral neuropathy with the mean of 58.9 years. The mean diabetes duration for the DM only group was 12.6 years and for diabetes mellitus with peripheral neuropathy (DMPN) group was 19.8 years. Ten participants with diabetes mellitus without neuropathy had a recorded foot deformity and none had a history of ulceration. One hundred and forty three DMPN participants had a recorded foot deformity and 29 had a history of ulceration. One study reported 2040 feet with deformity but did not subclassify them into having DM only or DMPN [26].

**TABLE 2:** Included papers and participant characteristics.

Groups	Healthy controls			Diabetes mellitus without peripheral neuropathy						Diabetes mellitus with peripheral neuropathy					
First author and year	Number of participants	Age (years) and gender (M/F)	Height (cm) and weight (kg)	Number of participants	Age (Years) and gender (M/F)	Height (cm) and weight (kg)	Type (1/2) and duration of DM (Years)	History of ulcer	Deformity present	Number of participants	Age (Years) and gender (M/F)	Height (cm) and weight (kg)	Type (1/2) duration DM (Years)	History of ulcer	Deformity present
Andersen et al., 2004 [32]	23	46 ± 16	179 ± 10	8	46 ± 35	176 ± 31	8	0	0	15	46 ± 35^a^	1.76 ± 31	15	0	0
		16/7	69 ± 20		16/7	73 ± 39^a^	27 ± 19				N/A	73 ± 39^a^	30 ± 17		
Bus et al., 2009 [17]	8	53.1 ± 9.2	174 ± 10.0	N/A						8	51.6 ± 11.1	178 ± 8.8	N/A	4	2
		6/2	84.3 ± 10.8								6/2		24.1 ± 9.8		
Bus et al., 2009 [17]	5	58.5 ± 7.1 or 58.7 ± 6.2 3/2^b^		N/A						20	(N + C+): 58.5 ± 7.1 6/4 (N + C−): 58.7 ± 6.2 6/4	(N + C+): 178 ± 8 83.3 ± 14.3 (N + C−): 173 ± 5 81.6 ± 10.2	(N + C+): 6/4 34.6 ± 11 (N + C−): 8/2 31.3 ± 14.6	0	10
Cheuy et al., 2013 [10, 18]	12	57 ± 14	174 ± 12	N/A						23	59 ± 10	173 ± 9	3/20	8	11
		8/4	108 ± 30								14/9	109 ± 27	18 ± 10		
Cheuy et al., 2016 [20]	N/A			N/A						34	59 ± 10	170 ± 9	0/34	0	34
											14/9	108 ± 19	18 ± 10		
Hastings et al., 2016 [29]	12	57 ± 14	174 ± 12	N/A						23	59 ± 10	173 ± 9	3/20	0	23
		8/4	108 ± 30								14/9	108 ± 27	17 ± 11		
Hastings et al., 2021 [28]	35	57 ± 14 8/4	174 ± 12 108 ± 30	N/A						23	(N + MCD−): 58 ± 8 8/4 (N + MCD+) 61 ± 12 6/5	(N + MCD−): 176 ± 9 110 ± 31 (N + MCD+) 171 ± 8 107 ± 31	(N + MCD−): 2/10 18 ± 10 (N + MCD+) 1/10 19 ± 11	N/A	11
Kimura et al., 2020 [19]	N/A			5	(N − C+): 59.9 ± 10.3 7/3 (N − C−): 56.3 ± 5.7 9/1	N/A BMI only: (N − C+): 34.5 ± 4.5 (N − C−): 33.9 ± 3.5	0/5 (N − C+) 8.0 ± 6.0 (N−C−): 4.8 ± 3.6	0	10	5	(N + C+): 60 ± 6.0 0/1 (N + C−): 57.2 ± 5.8 9/1	N/A BMI only (N + C+): 37.7 ± 4.6 (N + C−): 34.9 ± 8.3	0/5 (N + C+): 9.3 ± 3.7 (N + C−): 7.2 ± 5.3	0	10
Ledoux et al., 2003 [26]		N/A		1065	62.6 ± 10.6 1030/35	N/A	N/A 10.6 ± 9.5	0	2040^c^		N/A				
Robertson et al., 2002 [30]	16	57 ± 11	175 ± 12	N/A						16	56 ± 10	179 ± 10	5/11, 20 ± 11	16	16
		10/6	100 ± 36								11/5	104 ± 25			
Smith et al., 2000 [33]	1	40	183	N/A						1	65	183	N/A	1	1
		1	83.9								1	83.9			
Zellers et al., 2020 [22]	N/A			N/A						60	67 ± 6	N/A	0/60	0	60
											26/34	BMI only: 35 ± 7	19 ± 8		
Zellers et al., 2021 [31]	N/A			N/A						60	67 ± 6	168.8 ± 9.5	0/60	N/A	60
											26/34	99.6 ± 20.9	12 ± 13		

^a^ Did not differentiate between DM and DM with DPN.

^b^ For MRI reference only, age‐matched ± 5 years to experiment groups.

^c^ Number of Feet. Only considering hammer/claw toe deformity.

Abbreviations: (N + C−), neuropathy present, claw toes absent; (N + C+), neuropathy present, claw toes present; (N + MCD−), neuropathy present, medial column deformity absent; (N + MCD+), neuropathy present, medial column deformity present; (N − C−), neuropathy absent, claw toes absent; (N − C+), neuropathy absent, claw toes present.

### 4.4 Diabetic Neuropathy Measurements

Included measurement methods are demonstrated in Table 3. Nine papers tested for neuropathic status. Ten used the inability to detect a 5.07 (10g) Semmes–Weinstein monofilament at testing sites to determine neuropathic status [10, 17, 19, 20, 22, 27, 28, 29, 30, 31]. Three tested the motor nerve conduction velocity of the peroneal nerve [17, 27, 32]. Three used the Michigan neuropathy screening instrument (score > 2) to indicate the presence of neuropathy [22, 31, 32] and one used the neuropathy symptoms score [32].

**TABLE 3:** Included papers measurement methods.

First author and year	Diabetic peripheral neuropathy Measurement tool and method	Foot deformity Measurement tool and method	Intrinsic foot muscles Measurement tool and method
Andersen et al., 2004 [32]	Neuropathy symptoms score and neurological impairment score Electromyograph MNCV and VPT peroneal nerves	N/A	MRI non‐dominant foot: Muscle tissue separated; CSA and volume estimated using a stereological point counting method.
Bus et al., 2002 [27]	VPT at 5th MTH both feet Inability to sense 5.07 (10g) monofilament at minimum one test site MNCV peroneal nerve	Claw/Hammer toes Physical assessment non‐weight‐bearing measurements: TMTJ, MTPJ, PIPJ, DIPJ and tip of toe Joint angles calculated to determine toe joint configuration	MRI supine, both feet inserted into a head coil: Immobilised at 45° Multicomponent segmentation: Muscle and other tissue Normalised muscle CSA: Expressed as percentage of total foot CSA
Bus et al., 2009 [17]	VPT dorsal surface hallux both feet Inability to sense 5.07 (10g) monofilament at minimum one test site	Claw/Hammer toes MRI non‐weight bearing Toe angle: line parallel to sole of forefoot and bisector of proximal phalanx second or third toe (< −13° indicate extension deformity)	MRI supine foot into head coil: immobilised at 30° plantar flexion One image cutting through 5th MT head selected to score degree of IFM atrophy Semi‐quantitative 5‐point atrophy scale: 0‐healthy muscle, 4‐almost complete loss or complete loss of muscle
Cheuy et al. [10, 18]	VPT plantar surface great toe and first MT head. Inability to sense 5.07 (10g) monofilament at minimum one test site	Second MTPJ angle: Angle between longitudinal axis of second proximal phalanx and longitudinal axis of second MT parallel to dorsal cortex Medial column deformity Physical examination meeting at least 2 criteria: Calcaneal eversion ≥ 5°, medial longitudinal arch angle < 130°, navicular height ≤ 24 mm, medial column peak plantar pressure > 29 N/cm^2^	MRI supine, target foot perpendicular to table in head coil Edge detection algorithm for segmentation, separated into muscle and intermuscular adipose tissue (IMAT) volumes IFM deterioration: Ratio of IMAT volume to lean muscle volume of each MR slice averaged
Cheuy et al., 2016 [20]	VPT plantar surface both feet Inability to sense 5.07 (10g) monofilament MNSI score > 2	Claw/Hammer toes Physical examination visually most deformed second MTPJ alignment measured supine with ankle 30° plantar flexion MTPJ angle: The complement of the angle between the longitudinal axes of the second MT and second proximal phalanx	MRI supine, foot with most severe MTPJ deformity perpendicular to table in head coil Edge detection algorithm: lean muscle only and IMAT volumes Deterioration ratio: IMAT volume to lean muscle volume on each image
Hastings et al., 2016 [29]	VPT plantar foot surface Inability to sense 5.07 (10g) monofilament at minimum one test site	Meary's angle on lateral weight‐bearing radiographs: Angle formed by the line that bisects the talar head and neck and the line through the first MT longitudinal axis Medial column deformity measurements as described in [10, 18]	MRI supine, foot in head coil: IFM and fat volumes measured for all slices from talonavicular joint to tarsometatarsal joint Measurement methods as described in [10, 18]
Hastings et al., 2021 [28]	VPT plantar foot surface Inability to sense 5.07 (10g) monofilament at minimum one test site	Medial column deformity measurements as described in [10, 18]	MRI methods as described in [10, 18] Qualitative assessment of IFM fatty infiltration scores 0 (none) to 3 (severe) Categories included normal, mild atrophy (0–1, < 25% fatty infiltration of the muscle), moderate (2%, 26%–50%) and severe (3, > 50%).
Kimura et al., 2020 [19]	VPT plantar foot surface Inability to sense 5.07 (10g) monofilament at minimum one test site	Claw/hammer toes: Physical exam by single rater	CT bilateral neutral foot position, partial weight bearing 20% body weight IFMs segmented and volume calculated using mimics software
Ledoux et al., 2003 [26]	N/A	Claw/hammer toes: Physical exam by single rater	Physical examination: Muscle atrophy determined by palpating belly of extensor digitorum brevis by a specifically trained family nurse practitioner
Robertson et al. 2002 [30]	VPT plantar foot surface Inability to sense 5.07 (10g) monofilament at minimum one test site	Claw/hammer toes CT sitting with non‐weight‐bearing. MTPJ angle defined by subtracting the 180° angle formed by the intersection of metatarsal and proximal phalangeal lines	Axial CT images perpendicular to the long axis of the metatarsal Outline of plantar soft tissue superficial to the metatarsals and deep to dermis used as a surrogate measurement of plantar muscles
Smith et al., 2000 [33]	N/A	Claw/hammer toes CT seated non‐weight bearing and 50% weight bearing Hammer toe deformity represented by angle formed between metatarsal shaft and proximal phalanx	CT soft tissue thickness under mid‐shaft of each metatarsal measured Amount of fat and muscle, relative to plantar skin surface was measured orthogonal to metatarsal shaft for each location
Zellers et al., 2020 [22]	VPT plantar foot surface Inability to sense 5.07 (10g) monofilament at minimum one test site MNSI score > 2	Claw/Hammer toes CT positioned supine with ankle in 30° plantarflexion Second MTPJ angle measured as the angle formed by the long axis of the second MT and long axis of the proximal phalanx	MRI supine with extremity coil around foot Muscle and fat volumes segmented and measured as described in [10, 18] Muscle deterioration ratio (MDR): Inter/intramuscular adipose tissue volume divided by muscle volume
Zellers et al., 2021 [31]	VPT plantar foot surface Inability to sense 5.07 (10g) monofilament at minimum one test site MNSI score > 2	Claw/Hammer toes measurements as described in [22]	MRI supine with extremity coil around foot Muscle and fat volumes segmented and measured as described in [10, 18] Muscle deterioration ratio (MDR): Inter/intramuscular adipose tissue volume divided by muscle volume

Abbreviations: DIPJ, distal interphalangeal joint; MNCV, motor nerve conduction velocity; MNSI, Michigan neuropathy screening instrument; MTPJ, metatarsophalangeal joint; PIPJ, proximal interphalangeal joint; TMTJ, tarso‐metatartarsal joint; VPT, vibration perception threshold.

### 4.5 Intrinsic Foot Muscle Measurements

Nine studies used MRI, and three used CT (Table 3). One study used physical examination of the IFMs [26].

One study measured the intrinsic foot muscles as a whole [32]. Five papers measured all the IFMs from the forefoot to the hindfoot [10, 22, 28, 29, 31].

Three papers assessed the IFMs in the forefoot. Bus et al. [27] did not measure specific IFMs but performed a visual analysis of the IFMs at the level of the head of the fifth metatarsal [27]. Bus et al. [17], measured IFMs using an anatomically referenced coronal image cutting through the fifth metatarsal head but did not identify specific IFMs except the interossei and lumbricals [17, 20] and also did not measure specific muscles but performed a visual analysis of the IFMs of the forefoot from the second tarsometatarsal joint through to the sesamoids [20]. One paper measured the IFMs on the plantar surface of the foot [19]. One paper measured the Extensor Digitorum Brevis with palpation [26]. Two used the plantar soft tissue density under the midpart of the metatarsal shafts as a surrogate measurement for the IFMs using CT [30, 33]. Papers that included healthy controls all reported IFM atrophy and/or deterioration in comparison to DM and in greater degrees for DMPN participants.

### 4.6 Foot Deformity Measurements

Three studies investigated MCD, two with physical examination only [10, 28] and the other with physical examination and angular measurements on a lateral foot radiograph [29] (Table 3). Nine studies measured claw and/or hammer toe deformity using CT, MRI or physical assessment, with six only measuring MTPJ angle [17, 20, 22, 30, 33, 34]. Measurement software tools were variable; however, Zellers et al. [22, 31] and Hastings et al. [28] used the same MRI methods as Cheuy et al. [10]. One study used a Microscribe contact digitiser tool to measure both metatarsal and phalangeal joint angles [27]. Two studies used physical examination for classification [19, 26]. A specific metatarsal angle defining claw or hammer toe deformity was only mentioned in one study [17]; all other papers did not state what measurement angles represented deformity. In one study, no participant had claw/hammer toe deformity [32].

### 4.7 Associations Between Intrinsic Foot Muscles and Claw/Hammer Toe Deformity

Results and conclusions of the included papers are demonstrated in Table 4. For papers which provided *p* values a published guide was used to determine the strengths of these values, and in order to consistently compare *p* value strengths between papers. For correlation assessment, *r* values of 0–0.19 were considered very weak, 0.2–0.39 weak, 0.40–0.59 moderate, 0.6–0.79 strong and 0.8–1 very strong [35, 36]. One study reported a moderate positive correlation between IFMs and claw/hammer toe deformity measurements (*r* = 0.58, *p* = 0.02) [30]. Two studies reported a weak positive correlation (*r* = 0.273, *p* < 0.05) [22]; however, Kimura et al. [19] did not provide an *r* value [19]. Two papers reported moderate inverse correlations (*r* = −0.52, *p* < 0.01) [20] (*r* = −0.266, *p* = 0.043) [31]. One paper found a very weak inverse relationship between IFM atrophy and toe deformity using an observer determined muscle atrophy score (*r* = −0.18, *p* = 0.44) [17]. Overall, associations had conflicting results. Four papers measured both IFMs and foot deformity but did not assess for correlation [26, 27, 32, 33].

**TABLE 4:** Included papers results and conclusions.

First author and year	Results	Conclusions
Andersen et al., 2004 [32]	Intrinsic muscle volume (VFM) of DMN+ 49% ± 84 less than DMN− and controls (*p* < 0.001) DMN+: 86 ± 52 cm^3^ DMN−: 165 ± 34 cm^3^ Control: 168 ± 42 cm^3^ Inverse relationship between neuropathy rank‐sum score and VFM (*r* = −0.75, *p* < 1 × 10^5^)	No participant had significant foot or toe deformity Supports loss of foot muscles precedes development of toe abnormalities
Bus et al., 2002 [27]	73% decrease in IFM CSA of DMN+ compared to controls DMN+: 8.3 ± 8.1 42 cm^2^ Control: 30.8 ± 12.4 42 cm^2^ MTPJ angle not significant between groups DMN+: 46 ± 16°, two participants > 54° indicating claw/hammer toes Control: 45 ± 5°	Lack of toe deformity in majority of neuropathic subjects suggests that IFM atrophy is not a primary causative factor in toe deformity as formerly hypothesised
Bus et al., 2009 [17]	Correlation coefficient muscle imbalance score and toe angle not significant (*r* = −0.18, *p* = 0.44) 10% decrease muscle imbalance score N + C+ than controls N + C+: 2.2 ± 1.1 Control: 2.0 ± 1.0 17% decrease intrinsic muscle atrophy score N + C+ than controls N + C+: 3.1 ± 1.1 Control: 2.6 ± 1.2 85% decrease in MTPJ angle N+C+ than controls N + C+: −21.0 ± 5.6° Control: −3.1 ± 7.0°	Neither intrinsic muscle atrophy nor muscle imbalance was able to discriminate between neuropathic patients with claw toe deformity and those without
Cheuy et al., 2013 [10, 18]	57% reduction in IFM volume DMN+ compared to controls DMN+: 18.2 ± 11 cm^3^ Control: 31.6 ± 12.8 cm^3^ 52% increase in IFM adipose tissue volume DMN+ compared to controls DMN+: 17.9 ± 10.5 cm^3^ Control: 9.3 ± 3.8 cm^3^ MTPJ angle not significant between groups DMN+: 152 ± 11° Control: 153 ± 7° 400% increase (5×) of IFM ratio DMN+ compared to controls DMN+: 1.6 ± 1.2 Control: 0.3 ± 0.2 Negative correlation between IFM ratio and MTPJ angle (*r* = −0.51, *p* = 0.01)	Participants with diabetes and neuropathy have increased IFM deterioration, which was associated with second MTPJ angle hyperextension and history of ulceration
Cheuy et al., 2016 [20]	DMN + correlated with forefoot deterioration ratio (*r* = 0.53, *p* < 0.01) Forefoot lean muscle volume inversely associated with MTPJ hyperextension (*r* = −0.52, *p* < 0.01)	Lean muscle tissue volume of IFMs associated with severity of MTPJ deformity
Hastings et al., 2016 [29]	57% reduction in IFM volume compared to controls—Some data from [10, 18] DMN+: 18 ± 11 cm^3^ Control: 32 ± 12.8 cm^3^ 52% increase in IFM adipose tissue volume DMN+ compared to controls DMN+: 17.9 ± 10.5 cm^3^ Control: 9.3 ± 3.8 cm^3^ 18% decrease in Meary's angle DMN+ compared to controls DMN+: −11 ± 5° Control: −13 ± 11° IFM volume correlated with Meary's angle (*r* = 0.51, *p* = 0.01) IFM adipose tissue volume not correlated with Meary's angle (*r* = 0.08, *p* = 0.01)	Muscle deterioration and tendon deterioration are predictive of medial column foot deformity and poor midfoot function in individuals with DMN+
Hastings et al., 2021 [28]	20% increase in MRI qualitative score (IFM fat infiltration) DMN + MCD + compared to DMN + MCD− and 450% compared to controls. DMN + MCD+: 2.09 ± 1.05 DMN + MCD−: 1.58 ± 1.17 Control: 0.25 ± 0.45 27% decrease in Meary's angle DMN + MCD+ compared to DMN + MCD− and 5× increase compared to controls. DMN + MCD+: −36° DMN − MCD−: −10° Control: −4° Muscle scores were not significantly correlated with any radiographic alignment measures (no *r* or *p* values given)	Did not find a relationship between qualitative examinations or MR images of soft tissue deterioration and medial column deformity
Kimura et al., 2020 [19]	40% decrease intrinsic muscle volume N + C+ compared to N + C−, 10% decrease N − C+ compared to N − C − N + C+: 69.30 ± 8.87 N + C−: 109.80 ± 8.63 N − C+: 104.60 ± 8.64 N − C−: 114.40 ± 8.83 Correlation between intrinsic muscle, neuropathy, and claw toe status *p* = 0.083 (no *r* value given) Presence of neuropathy was significantly associated with decreased IFM volume for those with claw toes	Neuropathic feet with claw toes have decreased intrinsic foot muscles; likely the effect of neuropathy and claw toes is synergistic Intrinsic muscle atrophy increased plantar aponeurosis thickness, and neuropathy are all potentially related to the complex the development of claw toe deformity.
Ledoux et al., 2003 [26]	56% increase in IFM atrophy for pes cavus and 45% for pes planus feet compared to neutrally aligned Neutrally aligned: 49% Pes cavus: 78.1% Pes planus rigid: 74.4% Pes planus flexible: 67.7% 43% increase in hammer/claw toes for pes cavus and 23% for pes planus compared to neutrally aligned Neutrally aligned: 60.4% Pes cavus: 86.4% Pes planus rigid: 74.7% Pes planus flexible: 72.6%	Pes cavus feet demonstrated increased frequencies of bony prominences, claw/hammer toes, and prominent metatarsal heads Neutrally aligned feet have significantly lower frequency of intrinsic foot muscle atrophy, bony prominences and claw/hammer toes
Robertson et al., 2002 [30]	No difference in plantar soft tissue thickness beneath the metatarsal heads between diabetic and control group DM: 10 ± 2 Control: 10 ± 1 27% increase in MTPJ angle in the first toe compared to control (*p* = 0.04) and 36% increase in the third toe (*p* = 0.029) First toe DM: 27 ± 21 Control: 10 ± 5 3rd toe DM: 45 ± 16 Control: 33 ± 12 For individuals with diabetes MTPJ inversely correlated with plantar soft tissue density (*r* = −0.46, *p* = 0.07) 14% decrease in MTPJ arthropathy score those with diabetes compared to control *p* < 0.01 DM: 51.5 ± 7.8 Control: 58.6 ± 2.5 For individuals with diabetes MTPJ arthropathy score inversely related to MTPJ angle (*r* = −0.37, *p* = 0.001) and directly related to soft tissue density (*r* = 0.58, *p* = 0.02)	Extension of MTPJ more prevalent among individuals with diabetes, likely as a result of muscle wasting produced by diabetes associated neuropathy
Smith et al., 2000 [33]	Inconsistent differences in plantar soft tissue thickness beneath the metatarsal heads between diabetic and control group DM First MT DMN+: 9.0 mm, 3.5 mm weighted Control: 10.1 mm, 3.5 mm weighted Second MT DMN+: 14.0 mm, 7.5 mm weighted Control: 8.0 mm, 5.5 mm weighted 32% increase in first metatarsal angle DM subject and 55% weighted DMN+: 57°, 45° weighted Control: 43°, 25° weighted Wasting of intrinsic muscles visually evident in DMN + subject	Most severe hammer toe deformity in subject with DM was in second toe, which corresponds to ulcer under second MT head Wasting of intrinsic muscles was evident in the subject with DMN+
Zellers et al., 2020 [22]	DMN + participants only with MTPJ deformity Second MTPJ angle: 53 ± 12.8° IFM deterioration ratio: 0.32 ± 0.18 Weak positive correlation between second MTPJ angle and IFM deterioration ratio (*r* = 0.273, *p* < 0.05)	Poor IFM quality, impaired ankle dorsiflexion range of motion and greater ABI demonstrated weak to moderate relationships with greater MTPJ deformity
Zellers et al., 2021 [31]	DMN + participants only with MTPJ deformity Michigan score 5 ± 2 Second MTPJ angle 53 ± 12.8° Intrinsic muscle deterioration ratio 0.26 ± 0.14 Toe extension movement pattern (mean all tasks): 30 ± 10 Michigan score inverse correlation to toe‐extension movement pattern (*r* = −0.373, *p* = 0.003) IFM deterioration ratio inverse correlation to toe‐extension movement pattern (*r* = −0.266, *p* = 0.043)	Higher neuropathy severity and poor intrinsic foot muscle deterioration ratio has weak to moderate relationships with low toe‐extension movement pattern variability

Abbreviations: DMN−, diabetes mellitus, neuropathy absent; DMN+, diabetes mellitus, neuropathy present; DMN + MCD−, diabetes mellitus, medial column deformity absent; DMN + MCD+, diabetes mellitus, medial column deformity present.

### 4.8 Medial Column Deformity

Two papers found a moderate inverse correlation between IFMs and MCD measurements; one positive (*r* = 0.51, *p* = 0.01) [29] and one inverse (*r* = −0.51, *p* = 0.01) [10] (Table 4). One stated that muscle scores were not significantly correlated with any radiographic alignment measures; however, no *r* or *p* values were provided [28].

## 5 Discussion

### 5.1 Introduction

The primary aim of this systematic review was to investigate the relationships between the IFMs and diabetic foot deformity. Despite some conflicting results, most studies found an association between IFM atrophy or deterioration and foot deformity. However, they were generally weak, or only significant for participants with severe muscular atrophy. A secondary aim was to explore the methods used to investigate this relationship; many measurement variations were reported.

### 5.2 Associations Between Intrinsic Foot Muscles and Claw/Hammer Toe Deformity

Six of the 13 papers found either weak to moderate association between IFM atrophy or degradation and claw/hammer toe deformity [17, 19, 20, 22, 30, 31] (Table 4). Four papers measured both claw/hammer toe deformity and IFMs but did not conduct a statistical analysis for correlations [26, 27, 32, 33].

Despite an overall low to moderate association, some papers found that correlations became more significant in certain conditions. Robertson et al. [30] observed that plantar foot muscle density was related to the bone density of the metatarsal heads [30]. Kimura et al. [19] found the presence of neuropathy was significantly associated with lower IFM volume only for those with claw toes [19]. Similarly, Zellers et al. [31] found the Michigan score and IFM deterioration combined accounted for 18% of toe extension variability [31]. This supports a more synergistic or additive model of IFMs and claw/hammer toe deformity.

The original theory of muscle imbalance resulting in foot deformity assumes, without experimental evidence, that atrophy results in weakness of the muscle. However, a study examining the reliability and correlates of the cross‐sectional area (CSA) of Abductor Hallucis measured by US found a significant positive correlation between CSA of Abductor Hallucis and toe flexion strength measured by pedobarography (*r* = 0.562 *p* = 0.012) supporting the idea that CSA is a surrogate measure of muscle strength [37]. A recent study by Kaszyk et al. [38] has reported that IFM volumes were correlated with midfoot function in gait in those with diabetic peripheral neuropathy. In this study, decreased total muscle volume measured with CT correlated with greater midfoot collapse during gait (*r* = −0.40, *p* = 0.02) [38].

In addition, the theory of imbalance states that the extensor digitorum longus is the primary antagonistic muscle against the IFMs resulting in claw and hammer toe deformity. Bus et al. [17] provides experimental evidence against this theory; however, their methods did not accurately measure these involved muscle groups. IFM atrophy was determined using one image slice through the fifth metatarsal head in the coronal plane and a proximal and distal score of the extensor digitorum longus to determine an extrinsic foot muscle atrophy score. However, reliability of the extensor digitorum longus atrophy score was not reported and the use of the single image to determine IFM atrophy excludes the intrinsic foot muscles acting on the fifth toe and the muscle bellies of the flexor hallucis brevis and adductor hallucis, and omits the largest CSA of these muscles which are typically more distal in the forefoot [39, 40]. In addition, given significant reduction of IFM CSAs are reported due to advancing age [41] it is possible that age influences the atrophy scores in Bus's [17] study, not just health status.

Thus, the theory of muscle imbalance of the extrinsic versus intrinsic foot muscles is at best simplistic. Factors influencing the development of claw toes in patients with diabetes is likely complex and includes IFM atrophy, poor intrinsic and extrinsic muscle sequencing, and compromised extensor apparatus of the foot [42]. More specifically, toe clawing may involve extensor digitorum longus contractures causing MTPJ extension. In addition, extensor digitorum brevis [43], lumbricals and interossei muscles can atrophy, limiting their ability to stabilise the MTPJs in a neutral position. This is exacerbated by muscle deterioration, as evidenced by reduced volume and size in plantar IFMs [19, 20, 39]. This compromises flexion and control of the proximal interphalangeal joints. Additionally, the flexor digitorum longus is likely to have contractures as part of the flexor apparatus of the foot [44]. Shortening of the flexor digitorum longus contributes to interphalangeal joint flexion and toe clawing as it attaches to the phalanges more distally than the flexor digitorum brevis, thereby straining the MTPJ stabilising ligaments of the extensor apparatus. This demonstrates the complexity of possible mechanisms that may cause clawed toes in those with DPN [42, 45].

Diabetic foot deformity is a multifactorial disease. Three papers reported that ankle joint mobility is associated with claw/hammer toe deformity [20, 22, 29]. Insufficient stabilisation of the MTPJ by the IFMs may cause the extensor digitorum longus to shorten and produce hyperextension of the MTPJ, increasing reliance on the extrinsic foot muscles for gait and affecting ankle joint mobility [20]. Bus et al. [17] described how pathology of the plantar aponeurosis contributes to MTPJ instability and hyperextension of the plantar aponeurosis. Zellers also found that bone mineral density is significantly associated with MTPJ deformity [22]. Kimura et al. [19] found a significant interaction between increased plantar aponeurosis thickness, neuropathy and claw toe status. Therefore, these additional factors should be considered alongside the currently accepted factors that contribute to the development of foot deformity, including ligament contraction, inappropriate footwear and foot care.

An earlier literature review assessed three studies that investigated associations between IFMs and foot deformity [21]. They reported limited to no associations, whereas our review supports a weak to moderate association between IFM atrophy and the development of foot deformity. This may be due to the complex interactions between the extrinsic and IFMs.

### 5.3 Associations Between Intrinsic Foot Muscles and Medial Column Deformity

Cheuy et al. [18] recruited participants with MCD and found the correlation between MTPJ angle and IFM deterioration becomes more linear with increasing deterioration (Table 4). They suggest that a threshold effect, occurring at equal parts fat and muscle in the IFM compartment, is significantly associated with deformity.

Two studies, despite using the same measurement methods for IFMs and foot deformity found differing results; one study reported IFM deterioration and posterior tibialis tendon pathology were key predictors of medial column alignment and poor midfoot function in individuals with DMPN [29]. In contrast, Hastings et al. [28] found that DMPN + MCD had mild to moderate fatty atrophy of the IFMs, but not significantly correlated to MCD. They found that a higher total bone score, including bone marrow oedema, new bone formation and subchondral cysts, was associated with increased severity of medial column deformity MCD [28]. It is unclear why there are conflicting results despite the same participant demographics and measurement methods.

## 6 Imaging Modalities—Intrinsic Foot Muscles

MRI was used to investigate the IFMs for nine of the 13 papers and is optimal in evaluating soft tissue with high spatial resolution and image quality [46] (Table 3). One study reliably quantified intermuscular adipose tissue (IMAT) volumes, including muscle and fat segmentation, with most interclass correlations (ICCs) over 0.95 and validity of *R* ^2^ > 0.97 for all phantoms in their study [18]. MRI is an ideal choice for imaging the IFMs; however, there are contraindications for some patients with metal implants and claustrophobia, alongside high costs and long scanning times [46].

CT scans discriminate bone and muscle well, making it another appropriate tool for the assessment of IFMs [47]. One CT study [30] used the soft tissue density under the metatarsal shafts as a surrogate measure of IFMs, as there was difficulty in visualising and separating them from surrounding fat and tendons. Another study [22] used CT as a measurement tool and segmented volumes for more accurate and reliable measurements of IFM atrophy [48]. However, CT may not be considered suitable as a screening tool because of the high levels of radiation dosage and relatively high cost [46].

Ledoux et al. [26, 26] used physical examination of the extensor digitorum brevis as a measure of IFM atrophy [49]. They found that atrophy can rarely be diagnosed unless the extensor digitorum brevis is completely or nearly absent. Despite the test being easy to perform, it does not provide a comprehensive assessment of the IFMs and has subjective bias, which reduces accuracy of results.

None of the included papers used ultrasound (US) to measure or assess atrophy or degradation of the IFMs. Atrophy of the IFMs determined by ultrasonography has been validated against MRI measurements [43]. Similarly, in a separate study measuring IFMs, Wang et al. [50] used high frequency US to show significant IFM atrophy in diabetic feet [50]. US has considerable promise as a screening tool for diabetic foot management as the benefits of no ionising radiation, low cost and widespread accessibility potentially outweigh lower spatial resolution and operator dependency [46].

## 7 Imaging Modalities—Claw/Hammer Toe Deformity Measurements

The optimal modality for assessing musculoskeletal deformity is CT due to its superior cortical bone contrast and spatial resolution [46]. It can provide accurate and precise foot measurements for diabetic feet, enabling descriptions of internal and external structures [51]. However, the limitations of radiation dosage and relatively high cost apply.

The most common measurement of toe extension deformity by CT was the MTPJ angle [20, 22, 30, 33]. Despite wide acceptance as a measure of the severity of claw and hammer toe deformity, with low bias and high reliability [52] (Table 3), MTPJ angle does not distinguish between claw and hammer toe deformity. This is because the proximal and interphalangeal joint angles are not assessed for flexion and extension and is a drawback to understanding the causes and progression of these deformities.

Bus et al. [27], was able to measure MTPJs, PIPJs and DIPJs using a Microscribe 3D digitiser, which was potentially able to provide a greater assessment of all the toe joint angles. Even though CT measurements are considered the gold standard, 3D digitiser measures of MTPJ angle have been found to be highly correlated with CT measures, and are reliable and validated radiological measures [53].

## 8 Imaging Modalities—Medial Column Deformity Measurements

Two papers [10, 29], utilised plain radiographs for angular measurements in assessing foot deformity (Table 3). However, this may not be as reliable as CT, providing only a two‐dimensional representation with inherent superimposition of bony anatomy [10].

One study, Cheuy et al. [10], used validated references for defining the parameters of MCD. However, the main measurements of the study focused on MTPJ extension deformity [10]. Although MTPJ angle is the most common measurement of assessing claw or hammer toe deformity [52], there was no mention of or reference to these deformities in the paper. Although all participants had an MCD, this paper focused on MTPJ hyperextension deformity, potentially representing claw/hammer toe deformity.

Hastings et al. [29], investigated specific measurements regarding MCD [10], focusing on Meary's angle. This has been found to have lower precision than other radiographic bony measurements of foot alignment due to difficulties in measuring the talus [54]. This is important as Meary's angle is defined as the angle formed by the line bisecting the talar head and neck and the line through the longitudinal axis of the first metatarsal [29]. Yet, overall, it is an appropriate measure to assess MCD as part of a comprehensive assessment that examines medial and longitudinal arch alignment [55].

### 8.1 Limitations

All the papers included within this review were cross‐sectional studies thus a cause‐and‐effect relationship cannot be determined. All studies except Ledoux et al. [26] had small sample sizes, which may impact external validity and the power of the results. A meta‐analysis was also not possible, as the data were not sufficiently homogeneous. With all these factors, it is difficult to generalise results across all the papers as there were large variations in ages and demographic factors of participants. One of the most significant considerations is that of a wide age range of participants, as with older age, muscle size and quality deteriorates. Therefore, it is difficult to distinguish these changes from that of diabetes. Thus, a recommendation for future papers is to sub‐group participants into smaller age categories. Studies that were not in English language were not included; therefore, potential papers may have been missed.

### 8.2 Clinical Implications

This review has identified significant heterogeneity in the included publications, with weak associations between toe and foot deformity and muscle atrophy. Nonetheless, it has demonstrated that muscle atrophy is a likely contributor to structural changes in the diabetic foot. Clinically, since IFMs appear to contribute to the development of diabetic toe and foot deformity and play an integral role in foot function and gait, clinicians should encourage exercise programmes that target increasing the strength of the IFMs.

Additionally, although X‐ray and CT are useful for bony deformities, it seems ultrasound shows promise as a useful screening tool for the musculature of diabetic feet, given its portability, wide availability, and the capacity to be used in multiple clinical settings such as physiotherapy and podiatry. Clinicians may benefit from collaboration with their imaging colleagues or from learning the skill of ultrasound as a point‐of‐care application. In an era of restraint in its use, ultrasound has the advantage of no ionising radiation (Image Wisely; https://www.imagewisely.org/).

### 8.3 Future Recommendations

Future research focusing on factors that have a greater association with diabetic foot deformity is recommended, including bone mineral density, limited joint movement and plantar aponeurosis thickening. This will be beneficial in clarifying the development of diabetic foot deformity.

The use of US to investigate correlations between IFM atrophy and fatty infiltration due to diabetic foot deformity may be beneficial, providing real time information of muscle function in conjunction with ligament and tendon movements. Significant associations between IFM atrophy and lesser toe deformities determined by US has been reported in older adults with claw toes [39].

### 8.4 Conclusion

Despite recent advances, there is still limited understanding of the development of diabetic foot deformities. We found evidence that atrophy and increased intermuscular adipose tissue degradation of the intrinsic foot muscles are factors in the aetiology of these deformities. Currently, the imaging modalities of MRI and CT are the most accurate and reliable methods for investigating the severity of structural changes in the diabetic foot. Diabetic foot deformity is a complex, multifactorial complication of diabetes that should be prevented and managed in order to prevent ulcerations, infections, amputations and functional deficits.

## Author Contributions

**Tiffany Hanna:** conceptualization, data curation, formal analysis, investigation visualization, writing – original draft preparation, writing – review and editing. **Penelope Latey:** conceptualization, supervision, validation, methodology, project administration, writing – review and editing. **Kanchana Ekanayake:** data curation, investigation, resources, software. **Laura Ribarovski:** validation. **Rebecca McDonald:** validation. **Jillian Clarke:** conceptualization, resources, supervision, validation, methodology, project administration, writing – review and editing.

## Conflicts of Interest

The authors declare no conflicts of interest.

## Data Availability

Data sharing not applicable to this article as no datasets were generated or analysed during the current study.
